# Development of Tetrapod Zinc Oxide-Based UV Sensor for Precision Livestock Farming and Productivity

**DOI:** 10.3390/bios12100837

**Published:** 2022-10-07

**Authors:** Abbey Knoepfel, Na Liu, Yuchen Hou, Sathya Sujani, Barbara Roqueto dos Reis, Robin White, Kai Wang, Bed Poudel, Sanju Gupta, Shashank Priya

**Affiliations:** 1 Department of Materials Science and Engineering, Pennsylvania State University, University Park, PA 16802, USA; 2 Department of Animal and Poultry Sciences, Virginia Polytechnic Institute and State University, Blacksburg, VA 24061, USA

**Keywords:** tetrapod zinc oxide, UV sensor, space charge model, wearable sensor, ruminants

## Abstract

In order to ensure the health and welfare of livestock, there has been an emphasis on precision farming of ruminant animals. Monitoring the life index of ruminant animals is of importance for intelligent farming. Here, a wearable sensor for monitoring ultraviolet (UV) radiation is demonstrated to understand the effect of primary and secondary photosensitization on dairy animals. Thin films of wide bandgap semiconductor zinc oxide (ZnO) comprising multilevel of nanostructures from microparticles (MP) to nanoparticles (NP), and tetrapod (T–ZnO), were prepared as the UV sensing active materials. The sensitivity was evaluated by exposing the films to various radiation sources, i.e., 365 nm (UV A), 302 nm (UV B), and 254 nm (UV C), and measuring the electrical resistance change. T–ZnO is found to exhibit higher sensitivity and stable response (on/off) upon exposure to UV A and UV B radiation, which is attributed to their higher surface area, aspect ratio, porosity, and interconnective networks inducing a high density of chemical interaction sites and consequently improved photocurrent generation. A wearable sensor using T–ZnO is packaged and attached to a collar for dynamic monitoring of UV response on ruminant animals (e.g., sheep in this study). The excellent performance of T–ZnO wearable sensors for ruminant animals also holds the potential for a wider range of applications such as residential buildings and public spaces.

## 1. Introduction

Rapid industrialization and urbanization pose significant environmental challenges, as witnessed by increased concentration of pollutant gases in the atmosphere. Examples of these toxic gases include CO, SO_2_, NO_2_, H_2_S, CH_4_, and/or chemical and volatile organic compounds (VOCs) such as benzene, toluene, ethanol, acetaldehyde, and formaldehyde that are detrimental to living systems [1,2,3,4,5]. In particular, severe air pollution can cause abnormalities in lung surfactant composition, making living systems vulnerable to diseases [6]. Understanding the influence of these polluting gases and chemicals on both human health and the health of ruminant animals (e.g., cow, sheep, goats, and other cattle) is critical [7]. One of the key effects of pollution is increased ultraviolet (UV) exposure, which can induce photosensitization [8]. Primary photosensitization is a reversible condition where photo-cytotoxic compounds enter the bloodstream via the digestive tract. These compounds react in non-pigmented regions when exposed to UV radiation, causing cellular degradation and sloughing of the digestive chamber in the stomach of ruminant animals [9]. The effects of primary photosensitization can be mediated, however, by switching feed and keeping the ruminant out of sun for short spans [10]. Moreover, though the cow rumen is a very specific environment, improving our ability to study this ecosystem provides a notable opportunity to enhance understanding of fermentation, food production, and energy generation, not just within cattle but within anaerobic fermentation environments in general. Finally, secondary photosensitization is irreversible, as the liver is damaged due to the accumulation of plant and algae toxins, and increases the risk of developing skin cancer [10]. In order to mitigate losses due to photosensitization, a UV sensor is required to monitor the real-time radiation exposure of ruminants. A UV sensor will provide data on varying exposure levels at different farm locations (sunny and shaded) and guide the animal schedules around instances of the highest UV exposure at each of these locations. Non-invasive sensors allow for rapid detection and treatment of maladies in ruminants, improving the quality of animal life and decreasing any financial losses from lower productivity. There has been significant progress in design and development of UV sensors based upon metal oxides and semiconducting metal oxides [11,12] that have shown promise for UV sensing platforms.

Zinc oxide (ZnO) is a promising candidate for sensing UV light because it possesses a wide direct bandgap of 3.37 eV at room temperature, a large exciton binding energy of 60 meV (GaN has binding energy of 28 meV), n-type semiconducting behavior, and other unique physical properties which are attractive for optoelectronics devices such as UV photodetectors [13,14,15]. Compared with traditional infrared sensors, ultraviolet (UV) detectors have a higher signal-to-noise ratio and lower working temperature and have been widely applied in many areas, such as flame or gas sensing, UV astronomy, and secure communications [16]. Excellent sensing performance of ZnO is related to its resistance to photo-degradation along with its direct wide bandgap characteristics, catalytic properties, and ability to exhibit different morphologies [5,6,17]. ZnO can be synthesized into various morphologies and structures including micro-/nanoparticles, nanorods, nanoflowers, nanowires, and nanowalls [7,18,19]. ZnO morphology has been shown to play a significant role in controlling the sensing performance. For example, ZnO nanofiber- and nanowire-based sensors were found to display high responsivity toward UV radiation in ambient conditions [20,21].

Recently, ZnO tetrapod-shaped structures (T–ZnO) and their interconnected three-dimensional (3D) network have attracted attention for sensing capabilities due to their unique geometry and morphology. T–ZnO consists of four rod-like arms connected at tetrahedral angles to the spherical core, which is beneficial to form a large, mechanically flexible network with high porosity and high surface-to-volume ratio [6,7,8,22]. Zheng et al. fabricated a multi-terminal oxygen sensor based on an individual T–ZnO, which was capable of detecting light in different wavelengths and distinguishing false responses [22,23]. T–ZnO has also shown improved carrier mobility over zero-dimensional (0D) nanostructures, resulting in faster response and recovery times, comparable to those of one-dimensional (1D) nanostructures [8,24]. It also has a wide-ranging BET surface area, which can vary anywhere between 5 and 78 m^2^/g depending upon the morphological structure [25,26]. Thepnurat et al. fabricated UV sensors utilizing interconnected T–ZnO that showed promising results due to the linked tetrapod arms, which decrease the potential barrier at grain boundaries, thereby improving UV-induced charge carrier mobility [20]. Here, we focus on studying the UV sensing properties of the hybridized tetrapod structures of single and networked topologies. A ZnO UV sensor based on T–ZnO thin film is fabricated and its sensing performance is measured in terms of the electrical resistance change. The T–ZnO sensor performance is compared with that of microparticle (ZnO–MP) and nanoparticle ZnO (ZnO–NP) thin films, as well as their bulk counterpart. The results are discussed based on a space charge and grain boundary model, and band-bending theory. A two-terminal T–ZnO-based UV sensor is integrated onto a wearable collar to conduct field tests.

## 2. Experimental

### 2.1. Materials and Methods

#### 2.1.1. Preparation of T–ZnO and Corresponding ZnO-Based Thin Films

Tetrapod ZnO was prepared via a flame transport synthesis (FTS) method by utilizing zinc nanopowder with particle size of 40–60 nm (Sigma-Aldrich, St. Louis, MI, USA) and polyvinyl butyral (Sigma-Aldrich) as raw precursors [26,27]. As-synthesized T–ZnO powder was dispersed in dimethyl sulfoxide (DMSO) with concentration of 10 mg/mL and ultrasonicated for 2 h, followed by spin coating immediately onto cleaned commercial p-type <100> Si substrates of size 1.5 × 2.5 cm^2^. Spin coating was performed at 2000 rpm for 30 s and repeated eight times. In-between the coatings, the samples were placed on a 90 °C hot plate. Following spin coating, the samples were annealed at 500 °C for 2 h with a 4 °C/min heating rate. In order to synthesize ZnO–MP and ZnO–NP thin films, ZnO powder with particle size of <5 μm (MP) and 30–40 nm (NP) was purchased from Sigma-Aldrich and dispersed in ethanol with the same concentration as T–ZnO (10 mg/mL) and prepared using the same parameters as the T–ZnO. A bulk ceramic ZnO was also prepared from the T–ZnO and ZnO–MP powders by mechanically pressing the powder into a cylindrical alumina mold to form pellets followed by sintering at 1150 °C for 5 h with a 4 °C/min heating rate [28,29].

#### 2.1.2. Material Characterization and Fabrication of UV Sensor

All samples were characterized using X-ray diffraction (XRD) (PanAnalytical Empyrean III, Billerica-MA, USA) and scanning electron microscopy (SEM) (FEI Apreo S, Hillsboro, OR, USA) to investigate the crystallinity, phase distribution, and relative grain size distribution. XRD was performed in the 2θ range of 25–50° operating at 40 kV and 40 mA. UV–visible spectroscopy (Hitachi UH4150, Brisbane, CA, USA) was used to investigate the optical properties of the ZnO–MP, ZnO–NP, and T–ZnO precursor solutions and synthesized films. The electrodes were deposited on the films using conductive silver ink with resistivity of 5–6 μΩ cm (i.e., Ohmic contact) providing a ~10 mm (or 1 cm)-wide sensing area. Copper wires were connected to the electrodes for measuring of electrical properties. The responses of the films and sensing devices were measured using a Keithley 2401 electrometer (Cleveland, OH, USA) and KickStart Digital Multimeter software, v.2.8.0), in the dark and under a UV lamp in three distinct radiation wavelengths, i.e., 254 nm (UV C), 302 nm (UV B), and 365 nm (UV A) (Analytik Jena UVP 3UV Lamp, Tewksbury, MA, USA). The optical response was measured in an enclosure in the dark and under UV irradiation. The collar fabricated for field testing had a portable design and included a voltage data logger (Monarch Track-It Datalogger, Amherst-NH, USA) connected in series to a 1.18 MΩ resistor, and the T–ZnO UV sensor device was powered by a 3 V battery. The photoconductivity response was measured on ruminants in a dark barn under UV irradiation, in a partially shaded pasture, and in a fully sunny pasture, and the changes were determined in terms of resistance change (or sensitivity).

## 3. Results and Discussion

### 3.1. Structural and Morphological Characterization

**Figure 1:**
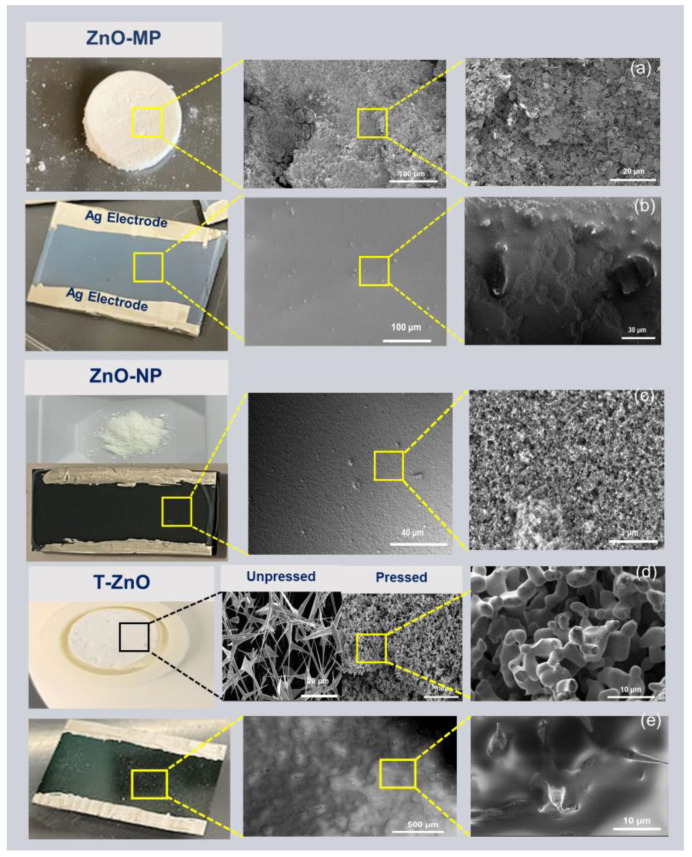
SEM images showing surface morphology. (**a**) ZnO–MP bulk pellet. (**b**) ZnO–MP thin film. (**c**) ZnO–NP powder and thin film. (**d**) T–ZnO pellet from the powder synthesized via direct flame transport method, unpressed and pressed forms showing networked tetrapod arms. (**e**) T–ZnO thin film. All of the thin films show colloidal silver (Ag) paste electrodes.

The morphology of all ZnO sample surfaces was obtained using field-emission SEM, as shown in Figure 1. Figure 1a and Figure 1d show the bulk pellet sample of ZnO–MP and T–ZnO, respectively, synthesized using the powder obtained through the direct flame transport method. Briefly, the ZnO–MP film (Figure 1b) and ZnO–NP film (Figure 1c) have uniform film surfaces. In comparison, the as-synthesized T–ZnO film displays a rough surface due to the generation of voids (Figure 1d). Figure 1d also displays the characteristic tetrapod feature, with arm length of ~9.7 μm. As for T–ZnO films, they produce an open network after deposition, showing individual tetrapods interconnected by the arms (Figure 1e). 

**Figure 2:**
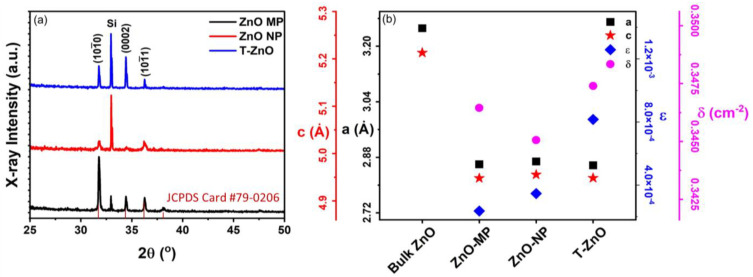
(**a**) XRD patterns of ZnO–MP, ZnO–NP, and T–ZnO thin films. (**b**) The calculated values of lattice constant, a, both from peak (002), lattice constant, c, from peak (101), lattice strain, ϵ, and dislocation density, δ. A reference for bulk ZnO XRD intensities (JCPDS Card #79-0206) is also included.

Figure 2 summarizes the X-ray diffraction (XRD) results (Figure 2a) as well as the analysis (Figure 2b) for ZnO–MP, ZnO–NP, and T–ZnO films. All ZnO-based thin films exhibited polycrystalline microstructure, having 2θ values with reflection planes occurring at 31.65°, 34.21°, 36.13°, and 47.34° corresponding to the lattice plane of (100), (002), (101), and (102), respectively (JCPDS Card No. 79-0206). These diffraction peaks can be indexed to those of hexagonal wurtzite ZnO structure. The peak at 2θ ≅ 32.91° is due to the Si (100) substrate [30]. The interplanar spacing for the ZnO (002) plane, d_002_, was calculated using Bragg’s Law of diffraction: 2dsinθ=nλ, where θ is the angle of diffraction with respect to atomic plane, λ is the X-ray wavelength (=1.5405 Å, for CuΚ_α_), and *n* is an integral number of wavelengths (=1 for first order diffraction) [31,32]. The average size of X-ray diffracting domains (XDD or crystallite size, L) was calculated following the Debye–Scherrer formula: L=Kλβcosθ, where *K* = 0.89 is Scherrer’s constant and β is the full width at half maximum (FWHM) of the (002) peak [33]. The L_002_ values for T–ZnO turns out to be 147.4 ± 10.5 nm, which is larger than the mean crystalline size for ZnO–MP (51.2 ± 6.3 nm), followed by ZnO–NP (22.5 ± 3.4 nm) [34]. The crystallite size is assumed to be the size of a coherently diffracting domain and it is not necessarily the same as particle size. The lattice constants, a and c, were calculated using: dhkl=[(43a2)(h2+k2+hk)+(l2c2)]−12 ;c=λsinθ, where θ = 17.20° for the (002) peak [35]. The lattice parameters were within 4% and 7% of bulk values (*ca. c* = 5.206 Å, *a* = 3.249, *c/a* = 1.602) and *c*/*a* = 1.71 ± 0.05 for all the films (see Figure 2b). We have also determined the dislocation density, δ, and lattice microscopic strain, ϵ, for all the films studied here. The dislocation density (δ) represents the defects associated with intrinsic stacking faults in the samples defined as the length of dislocation lines per unit volume of the crystal, and it can be calculated using δ = 1/L^2^, where L is the crystallite size [13,36]. The dislocation density (*δ*) is 7.8 × 10^−5^ (nm)^−2^, 2.9 × 10^−4^ (nm)^−2^, and 3.1 × 10^−4^ (nm)^−2^ for T–ZnO, ZnO–MP, and ZnO–NP films, respectively. Strain-induced broadening in powders and films due to crystal imperfection and distortion was calculated using the formula, ε = β_hkl_/4 tanθ [37,38], which is estimated to be 0.001, 0.002, and 0.003, for T–ZnO, ZnO–MP, and ZnO–NP films, respectively.

**Figure 3:**
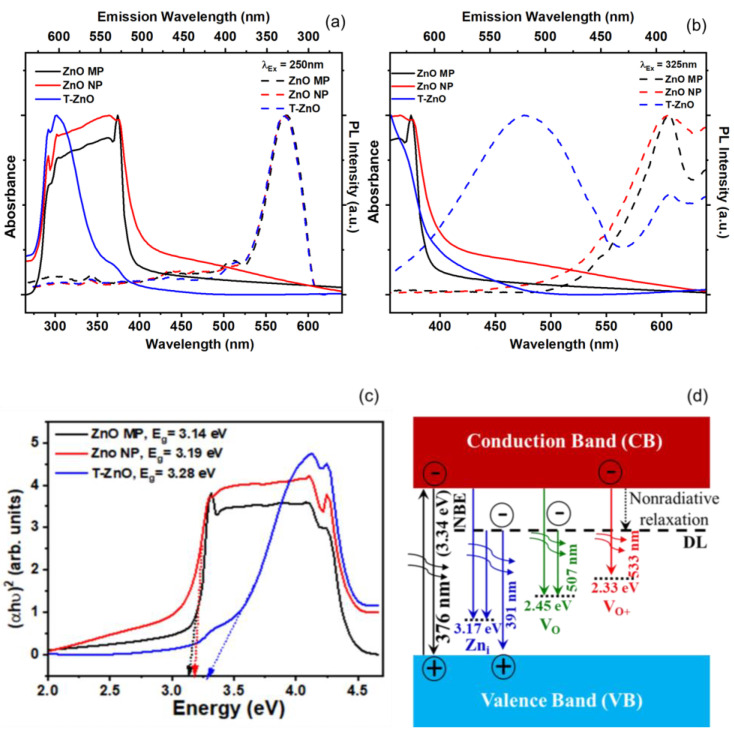
Optical spectroscopy of ZnO–MP, ZnO–NP, and T–ZnO. (**a**,**b**) UV–visible absorption spectra (absorbance) and photoluminescence (PL) spectra. (**c**) The calculated bandgap from Tauc gap. (**d**) energy levels schematic of near band edge. (NBE) and native point defects in ZnO lattice showing defect level (DL).

In order to observe the UV–Vis absorption spectroscopy of synthesized T–ZnO as well as that of ZnO–MP and ZnO–NP powder, they were sonicated in distilled water and ethanol for ~15 min, and absorbance (or optical density) was recorded. Figure 3 reports the absorption spectra for ZnO–MP, ZnO–NP, and T–ZnO (Figure 3a,b) along with a Tauc gap plot (Figure 3c) that is used to determine the bandgap for each of these ZnO materials. The absorption peak was recorded in each spectrum in range of 280–600 nm, which measures the characteristic band for the pure crystalline ZnO [39]. Absence of any other peak in spectra confirms that the synthesized materials did not have secondary structural phases. It is reported that the intensity of absorption peak in UV–visible spectrum is related to particle size of nanoparticles. The reduction in particle size is accompanied by bandgap increase, which requires a higher energy to excite electrons from the valence into the conduction band and results in the shift of the absorption edge [40,41,42]. While ZnO–MP and ZnO–NP produced a stronger peak at ~376 nm and ~375 nm, T–ZnO produced a larger magnitude peak at ~372 nm. The larger particle size of the T–ZnO (~10–20 μm arm length), compared to the aggregated ZnO–MP (<5 μm) and ZnO–NP (<100 nm), could factor into the observed comparable absorption peak magnitudes and being close to the bulk value (*ca.* bulk 368 nm). Investigating the optical and electronic properties of semiconductors, Tauc et al. [43,44] substantiated a method for determining the bandgap using optical absorbance data plotted appropriately with respect to energy. The absorbance (optical absorption strength) depends on the difference between the photon energy and the bandgap following this equation: (*αhν*)^1/^^*n*^ = *A* (*hν* − *E*_*g*_), where *h* is Planck’s constant, *ν* is the photon frequency, α is the absorption coefficient, *E_g_* is the bandgap, and *A* is a proportionality constant. Depending upon the nature of the electronic transition, whether allowed or forbidden and direct or indirect, is indicated by the value of exponent. Typically, the allowed transitions that dominate the basic absorption processes in semiconductors, giving either *n* = 1/2 or *n* = 2, for direct and indirect transitions, respectively. Plotting the (*αhν*)^2^ versus (*hν*) shown in Figure 3c for ZnO nanostructures provides a better fit and identifies the correct transition type yielding values of *E_g_* as 3.14 eV (ZnO–MP), 3.18 eV (ZnO–NP), and 3.28 eV (T–ZnO), respectively. The second power (*n* = 2) was used in these calculations as ZnO is well-known to have a direct transition [41]. Moreover, the characteristic features of Tauc plot are evident: at low photon energies, the absorption approaches zero, indicating that the material is transparent; near the bandgap value, the absorption becomes stronger and shows a region of linearity in this squared-exponent plot. This linear region extrapolated to the abscissa intercept provides the bandgap energy (*E_g_*) value. At even higher energies, the absorption processes saturate, and the curve again deviates from linear. On the low energy end, the deviation from linearity can be associated with defect absorption states that are near the band edge. 

In order to study the defect characteristics in ZnO samples, photoluminescence (PL) response was investigated with 250 nm and 325 nm excitation wavelengths, as shown in Figure 3a,b. The morphology and particle size are shown to influence the luminescence properties and show a co-existence of emission peaks near ultraviolet (violet and blue) and visible (green, yellow, and red) domains corresponding to excitons and point defects, respectively. The emission peaks, *albeit* weak, at 390, 460, and 560 nm are related to intrinsic oxygen vacancy (e.g., V_O_, V_O_^2+^) and interstitial zinc (Zn_i_) defect species [45,46,47]. However, under n-type conditions, where *E*_*F*_ is near the bottom of the conduction band, the oxygen vacancy is in the neutral charge state (V_O_). The photoluminescence result suggests the broad unstructured emission at ~2.21 eV (“green band”), which is attributed to the V_O_/_2+_ transition. The energy level band diagram schematic displaying characteristic ZnO transition and defect level (DL) emissions is provided as reference in Figure 3d. In general, oxygen vacancies are considered as green emission centers, whereas zinc interstitials are responsible for violet and blue emission. Other emissions due to zinc vacancies (V_Zn_) and O_i_ defects are located in the bandgap above the valence band and at times participate in the blue end of the emission spectrum.

### 3.2. Resistance Change Measurements in Controlled UV Environment

**Figure 4:**
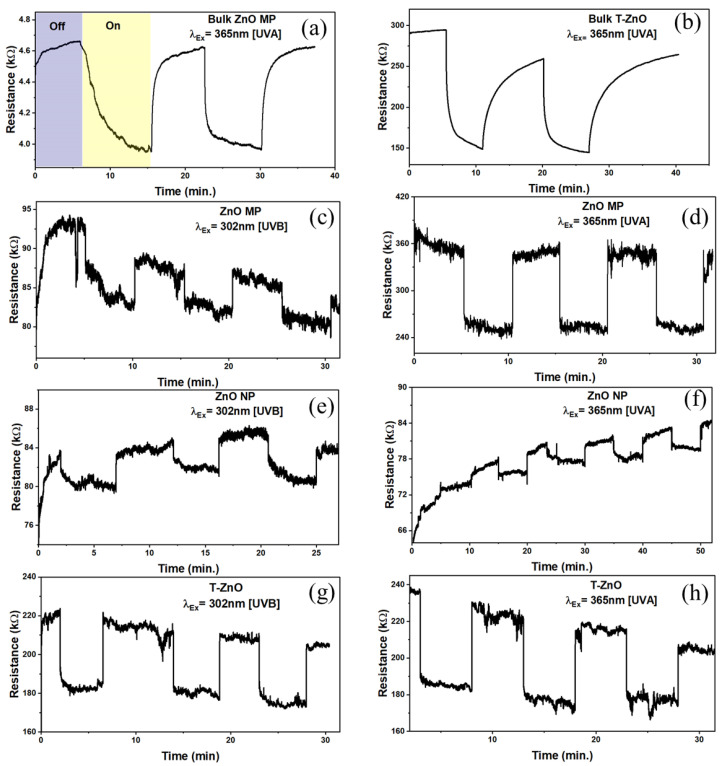
Resistance variation for bulk (**a**) ZnO–MP and (**b**) T–ZnO pellets in ambient environment exposed to UV A (365 nm) monochromatic light. Resistance measurements in enclosed environment exposed to UV A and UV B (302 nm) for (**c**,**d**) ZnO–MP, (**e**,**f**) ZnO–NP, and (**g**,**h**) T–ZnO.

ZnO is a promising II-VI semiconductor for visible–blind UV sensors in air because of its high radiation hardness [48,49] and versatile nanostructures [50]. It is well-known that the sensitivity is dominated by the dimensions, size, and shape of the microstructures. Response (on/off) ratio or sensitivity is one of the key parameters of UV (and gas) sensors and was determined from resistance changes in ZnO samples tested in a dark chamber upon UV illumination. The sensitivity was calculated as: S=ΔRR0=|RUV−Rdark|Rdark×100, where *R_UV_* was the maximum resistance measured when the sensor was exposed to UV radiation and *R_dark_* was the resistance measured prior to exposure of UV radiation [21]. Figure 4 shows the typical response curves for both the bulk ZnO–MP and T–ZnO as well as of thin films in terms of resistance in the dark and under two different UV illuminations (UV A, 365 nm and UV B, 302 nm) with an intensity of 1.4–1.6 mW/cm^2^. The T–ZnO films exhibited the highest sensitivity at both the 365 nm and 302 nm UV wavelengths (S = 39.21%). The sensitivity values under UVA illumination for ZnO–MP (S = 37%) and ZnO–NP (S = 35.8%) were closer to that of the T–ZnO, but the sensitivity values under UVB illumination for ZnO–MP (S = 15%) and ZnO–NP (S = 16.7%) were significantly lower compared to the T–ZnO films. The sensitivity values are summarized in Table 1 for all of the ZnO-based UV sensors excited at two different UV radiations. Besides the on/off resistance ratio, the sensors’ rise and decay times are used to characterize the time required for the resistance to rise from 20% to 80% of its final value, i.e., the steady-state photocurrent value. From the time-dependent response curves, in terms of resistance measurements by alternatively exposing the sensor to UV light (on) and dark (off) (Figure 4a–h), the rise (response) and decay (recovery) times were computed for 80% of the steady-state values, which are summarized in Table 1. T–ZnO films produced response (t_f_ = 5, 2 ms) and recovery (t_r_ = 2.4, 0.5 ms) times in an ambient environment (in air) at 365 nm and 302 nm wavelengths. The T–ZnO films produced comparable response (t_f_ = 3.3, 7.9 ms) and recovery (t_r_ = 0.5, 0.9 ms) times in an enclosed environment at 365 nm and 302 nm wavelengths to the ambient results, with better signal stability in the enclosed environment indicative of its high stability as a UV sensor. The ZnO–MP films had much faster response (t_f_ = 2.1 ms) and recovery (t_r_ = 0.8 ms) times compared to the ZnO–NP films (t_f_ = 372 ms, t_r_ = 3700 ms), comparable to T–ZnO films in both the ambient and the enclosed (Figure 4) environments at 365 nm wavelength (close to bandgap energy). The ZnO–MP films had significantly slower response (t_f_ = 15 ms) times at 302 nm wavelength (away from bandgap energy), which correlates with the UV–visible absorption spectra. This could be due to a denser microstructure produced by the repeated sintering process in T–ZnO and ZnO–MP. Bulk pellet samples of T–ZnO and ZnO MP were tested under 365 nm wavelength (Figure 4a,b) and produced response times (t_f, T–ZnO_ = 800 ms, t_f, ZnO–MP_ = 500 ms) and recovery times (t_r, T–ZnO_ = 4200 ms, t_r, ZnO–MP_ = 400 ms) similar to the ZnO–NP films, and were not mechanically stable as sensing platforms to develop wearable UV sensors [51]. The results show that the relatively long rise and decay times observed for ZnO–NP, and ZnO–MP, compared to T–ZnO, can be ascribed to the slow desorption of oxygen molecules from the ZnO films’ surface [52,53]. This is due to the open network of T–ZnO as well as interconnected arms, providing an efficient carrier path over the ZnO–MP and ZnO–NP films. The T–ZnO sensor was chosen for a wearable UV sensor for ruminants due to the consistent response and recovery times observed at both the 365 nm and 302 nm wavelengths.

**Figure 5:**
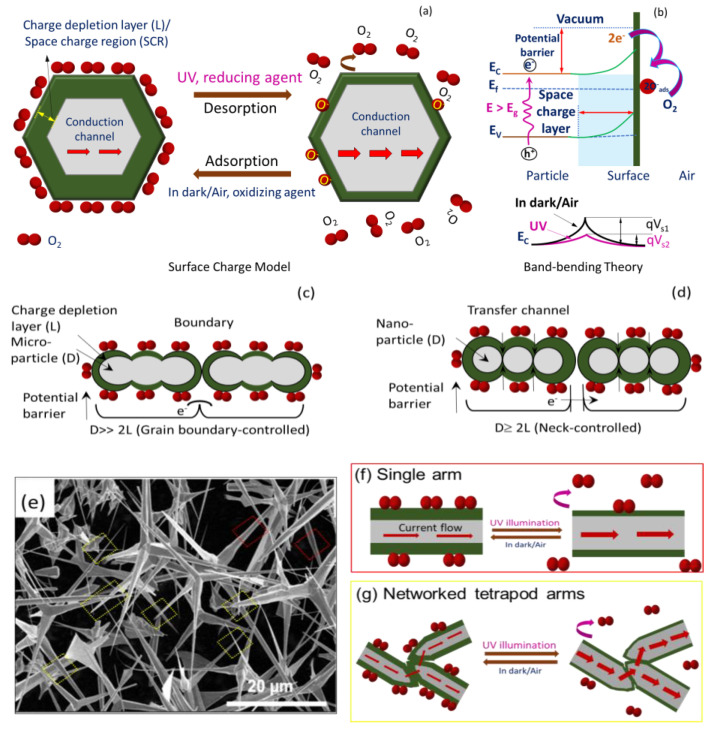
Schematic to describe sensing in dark/air and under UV illumination for ZnO using (**a**) space charge model and (**b**) band-bending theory. Proposed UV sensing mechanism based on space charge model for (**c**) microparticle ZnO–MP; D>> 2L, (**d**) nanoparticle ZnO–NP; D ≥ 2L. (**e**) SEM image of T–ZnO; D >> 2L and sensing mechanism of (**f**) single arm (red) and (**g**) networked tetrapod T–ZnO (D >> 2L) arm (yellow) morphology.

Briefly, ZnO is intrinsically an n-type (excess electrons as free carriers) semiconductor due to invariable presence of neutral oxygen vacancies (e.g., V_O_, V_O_^2+^) and interstitial Zn (e.g., Zn_i_^2+^, Zn_i_^+^, Zn_i_) as donor or compensating donor defects (see Figure 3d) [52]. The same characteristics are expected for T–ZnO as well as micro-/nanoparticles of ZnO. Since the formation energy of neutral oxygen vacancy is thermodynamically favorable, a majority of the oxygen vacancies remain in neutral state. Additionally, the resistive response of n-type ZnO sensor relies on the interaction between oxygen vacancies at the ZnO surface and charge accepting (or donating) adsorbate molecules [53]. Under low thermal activation (T ≤ 100 °C), molecular oxygen (O_2_) physi-sorbs onto the ZnO surface. Under UV illumination with photon energy higher than the ZnO bandgap of 3.37 eV (wavelength of less than 370 nm that are UV A and UV B), electron–hole (e–h) pairs are generated. Based on the ZnO sensor’s surface, when adsorbed molecular oxygen under UV activation captures photogenerated free electrons, they result in the chemisorption of oxygen ions to ZnO surface following: O_2_(g) + e^−^→O_2_^−^ (ads) or O_2_(g) + e^−^→2O^−^ (ads), leaving photo-induced holes in the low-conductivity depletion layer (or space charge region, SCR). According to the space charge model [25,54], there will be upward band-bending near the surface for both the conduction and valence bands shown in Figure 5a. As a result, there is an increase in energy barrier height for electron transfer between the grains or across neighboring T–ZnO arms, i.e., the qV_s1_, and thus the overall conductance is reduced (alternatively, increasing resistance). Notably, higher oxygen concentration can further bend the bands and lower the conductance at higher degree. As this process continues, the photo-generated holes migrate to the surface along the potential gradient produced by band-bending and react with chemisorbed oxygen species as h^+^ + O_2_^−^(ads) → O_2_(g), to discharge (desorb) molecular O_2_ from the ZnO surface. Consequently, the process leaves an excess of electrons in the conduction band of the lattice (lowering the Fermi level), and diminishes the energy of the depletion layer (reduced width), thus decreasing ZnO film resistivity [54]. Alternatively, it decreases the band-bending or barrier height so higher output current and conductance (low resistance) are observed, which is explained through band theory (i.e., qVs_2_ (UV) < qVs_1_), illustrated in Figure 5b [55]. Thus, the relative rise and decay times can be ascribed to the adsorption/desorption of oxygen molecules from the ZnO film’s surface. The rise time of sensors is related to the decreasing rate of the adsorbed oxygen ions (O^2–^). The decreased band-bending will slow down the holes’ migration speed, and result in a longer rise time. The decay time is determined by the rate of adsorbing O_2_ molecules to form O^2–^. After the UV light is turned off, the oxygen molecules will gradually be physisorbed on the ZnO, typically driven by the concentration gradient. As this physical–chemical process continues, the molecular oxygen density gradient near the ZnO surface decreases. The decreased oxygen density gradient will slow down the adsorption of oxygen, which can stretch or cause a longer decay time. 

Figure 5 provides the scheme of the UV sensing mechanism based on the charge depletion layer (or space charge region, L) (panel a) along with energy level diagram (panel b). In addition, the propensity of ZnO semiconductor particles of dimension D toward agglomeration, interconnectedness, and networked tetrapod arms are taken into consideration, such as in ZnO–MP (Figure 5c), ZnO–NP (Figure 5d), and T–ZnO (Figure 5f,g) sensor elements, which is also provided in Figure 5 along with the SEM image of T–ZnO (Figure 5e). Accordingly, since the semiconductor oxide particles are coagulated, the microstructure of these aggregates is considered to play a significant role in their functionality. Each of these ZnO particles in pressed pellet or thin films is connected with its neighbor either by grain boundary contacts, through arms, or by necks, as shown in Figure 5 for microparticles, tetrapods, and nanoparticles, respectively. While for microparticles and tetrapods (i.e., D >> 2L) the electrons should move across the surface potential barrier across each boundary, the electron transfer between nanoparticles (i.e., D ≥ 2L) takes place through a channel formed inside the space charge layer at each neck. The width of the channel is determined by neck size and L, and thus the sensitivity is dependent upon the particle size, D. It is worth noting that the sensitivity and, more importantly, the response and recovery times of T–ZnO, are superior because of the high aspect ratio of the microstructure, large active surface area, and networked arms. In all scenarios, the electrical resistance change, and hence the sensitivity, of the sensing element/analyte depends upon the microstructure of the sensing platform. Subsequently, it is well-known that the conductance of ZnO nanowires under UV illumination increases with little or no oxygen with higher signal-to-noise ratio [28]. The individual T–ZnO arms can be compared to ZnO nanowires, and as such exhibit a similar conduction response under UV illumination. Therefore, the ZnO-based UV sensing platforms developed in this work can be very well-expanded for oxygen under various UV illumination intensity [26].

### 3.3. Resistance Change Measurements on Ruminants

**Figure 6:**
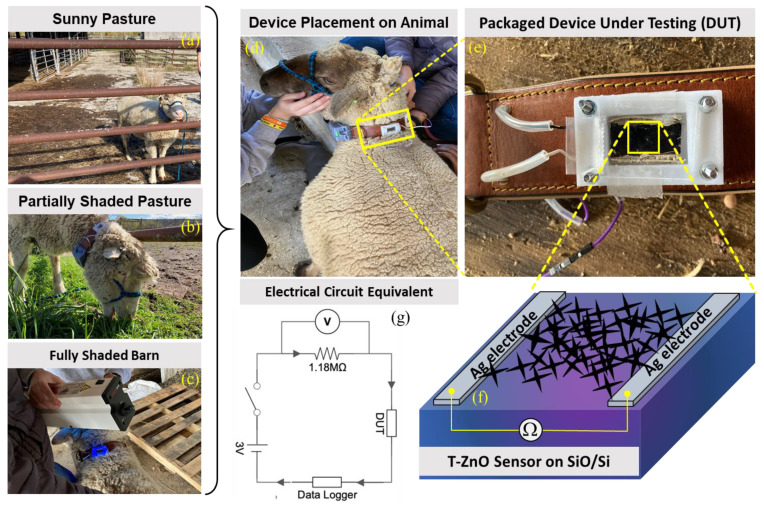
Wearable UV sensor fabricated from T–ZnO on ruminant (sheep) during testing under (**a**) sunny pasture, (**b**) partially shaded pasture, and (**c**) fully shaded barn with (**d**) attached data logger and (**e**) zoomed version, (**f**) schematic of device under test, and (**g**) an equivalent electrical circuit diagram.

**Figure 7:**
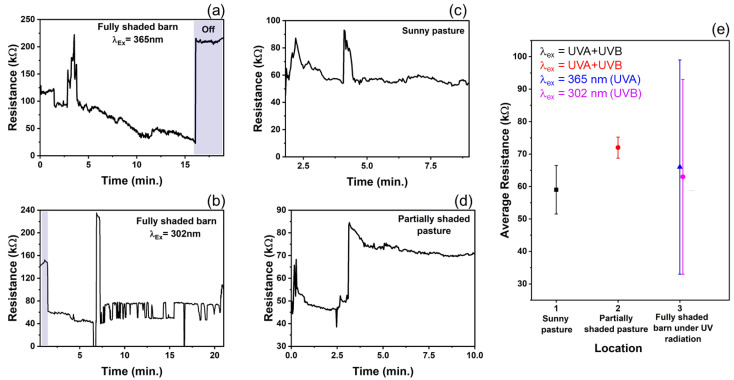
Resistance response of T–ZnO from collar placed on ruminant in various environments. (**a**,**b**) In dark barn under UV B and UV B irradiation (**c**) Full-sun pasture with UV light intensity ranged 2.2–3.0 mW/cm^2^, and (**d**) Partially shaded pasture with UV light intensity ranged 1.4–3.0 mW/cm^2^. (**e**) The average resistance of T–ZnO film from collar placed on ruminant, with standard error, in each respective environment.

In recent years, nanotechnology has received attention for improving livestock production [56]. In the U.S., only 26 of 160 agri-food nanotechnology research and development projects were relevant to livestock facilities [56]. Animal health, veterinary medicine, and other animal production facilities are a few of the livestock-related sectors on which nanoparticles (NPs) have their promising footprints. Therefore, based on the experimental findings and to expand the T–ZnO sensor’s adaptability and suitability, a wearable sensor was designed and deployed for animals’, especially ruminants’ (such as cow, sheep, goat, etc.) health monitoring and to prevent loss of productivity. T–ZnO thin films were used to measure resistance changes as a wearable UV light sensor due to the consistent results at both the UV illumination wavelengths. The device design and equivalent electrical circuit diagram are shown in Figure 6. The T–ZnO films were tested in the packaged device attached to the ruminant under 365 nm wavelength UV irradiation and produced response times consistent with values measured in controlled environments (5.6 ms on ruminant vs. 3.3 ms in controlled environment) and recovery times higher than values measured in controlled environments (9.8 ms versus 0.5 ms). The T–ZnO films tested under 302 nm wavelength UV irradiation had higher response (27 ms versus 7.9 ms) and recovery times (13 ms versus 0.9 ms) than the films measured in controlled environments. The variation in the response and recovery times observed for the T–ZnO film on the ruminant can be attributed to the changing intensity of the UV illumination and the distance between the UV light and the device changing as the ruminant moved, while the distance between the T–ZnO films and the UV light source was fixed in the controlled environments. The T–ZnO film-based sensor was tested while the ruminant animal (e.g., sheep) used in this study was grazing in a partially shaded pasture, fully shaded barn, and a full-sun pasture. All of the raw data (see Figure 7a–d) and the analysis of these measurements are presented in Figure 7e. The resistance in the fully shaded barn under 365 nm UV illumination decreased to ~33 kΩ (Figure 7a). Upon removing the UV illumination, the resistance increased to ~220 kΩ. The peak observed at 3.5 min was due to the ruminant moving its head away from the UV light source. Similarly, the resistance in the fully shaded barn under 302 nm UV illumination leveled out at ~71 kΩ (Figure 7b), and the peak observed at 7 min was also due to the ruminant moving away from the UV light source, resulting in larger error margins for the average resistances in the fully shaded barn, as in Figure 7e. The slower recovery time could be attributed to the barn not being completely dark. The resistance values in the full-sun pasture leveled out at ~57 kΩ (Figure 7c). The peaks observed at 2.5 min and 4.75 min were due to the collar rotating to the underside of the ruminant’s neck, which caused the resistance to increase. The resistance values increased by ~30 kΩ in the partially shaded pasture when the ruminant moved to the shaded region (Figure 7d). This brief study demonstrates that the resistance of T–ZnO films changes in response to changing UV exposure due to the UV sensing mechanism detailed above. The largest change in resistance of the T–ZnO film was observed in the fully shaded barn under direct UV illumination, while the changes in resistance were smaller when measured in the partially shaded and full-sun pastures. More in-depth studies are necessary to quantify the relationship between UV exposure and changes in the resistance of T–ZnO films. However, qualitative conclusions can be drawn, and appropriate preventative measures can be taken to minimize ruminants’ UV exposure based on the T–ZnO sensing device. We continue to improve our sensor platform by expanding the ZnO morphologies and integrated device design stability for animal health monitoring as well as agricultural applications.

## 4. Conclusions

In conclusion, we prepared a sensitive UV sensor based on T–ZnO and compared with other microstructures including ZnO micro-/nanoparticles. Our results clearly indicate the increased sensitivity, faster response, and recovery times for T–ZnO featuring a morphology with high specific surface area due to their open structure and interconnected and networked arms. We provided insightful discussion into the UV sensing mechanism through conceptual models for all the morphologies, signifying the importance of microstructure required for efficient performance. Subsequently, we used T–ZnO films to fabricate a wearable UV sensor and deployed for livestock (especially sheep) monitoring to demonstrate a proof-of-concept principle. The outcome from this study is applicable for the agricultural sector employing T–ZnO based sensors for monitoring other ruminant animals such as cows, especially for sensing enteric methane (CH_4_) and exploring ways to mitigate such emissions from ruminants. We plan to improve sensor measurement stability by developing a robust collar design and remotely controlled monitoring. The morphology and high surface area of the resulting T–ZnO structures will be advantageous in the development of devices for wide-ranging applications in complicated microenvironment observations, including chemical gas sensors, catalysis and photocatalysts, and animal fluids.

## Figures and Tables

**Table 1:** Summary of sensitivity, response time, and recovery time for various ZnO morphologies and microstructures tested in this study.

	λ_ex_	Sensitivity (Δ*R*/*R_dark_*) ^a^	Response Time, t_r_^b^	Recovery Time, t_r_ ^b^
Bulk ZnO MP	365 nm	15%	0.5 s	0.4 s
Bulk T–ZnO	365 nm	43.80%	0.8 s	4.2 s
**Spin–coating**				
ZnO–MP	302 nm	15%	15 ms	0.5 ms
	365 nm	37%	2.1 ms	0.8 ms
ZnO–NP	302 nm	16.7%	372 ms	3700 ms
	365 nm	35.8%	1600 ms	99.4 ms
T–ZnO	302 nm	21%	7.9 ms	0.9 ms
	365 nm	39%	3.3 ms	0.5 ms
**Drop-casting**				
ZnO–NP+PVB	302 nm	3.5%	39 s	18.5 s
	365 nm	3.99%	0.11 s	0.282 s
T–ZnO+PVB	302 nm	13.7%	11.1 s	11.1 s
	365 nm	23.2%	0.106 s	0.106 s

^a^ Defined as |*R_UV_* − *R_dark_*|/*R_dark_*; ^b^ Defined as increasing or decreasing resistance by 80%.

## Data Availability

Not applicable.
